# Comparison of milk and grass composition from grazing Irish dairy herds with and without milk fat depression

**DOI:** 10.1186/s13620-023-00230-3

**Published:** 2023-02-27

**Authors:** O. B. Neville, A. G. Fahey, F. J. Mulligan

**Affiliations:** 1 School of Veterinary Medicine, University College Dublin, Belfield, Dublin 4 Ireland; 2 School of Agriculture and Food Science, University College Dublin, Belfield, Dublin 4 Ireland

**Keywords:** Milk fat, Fatty acids, Perennial ryegrass, Dairy cow, Grazing

## Abstract

**Background:**

This study investigated the factors relating to pasture chemical and fatty acid (FA) composition that influence the milk fat percentage of spring calving, grazing dairy cows. The relationship between milk fat percentage and FA composition of the milk in these herds was also investigated.

**Results:**

Milk protein percentage, milk casein percentage and cheddar cheese yield were increased in milk from HMF herds. Cows from LMF herds did not have negatively altered milk processability including rennet coagulation time (RCT), pH and ethanol stability. Crude protein, NDF, ADF, ether extract and total FA content of pasture was not different between LMF and HMF herds. Milk fat concentration of conjugated linoleic acid (CLA) t10, c12 was not different between HMF and LMF herds. Pre-grazing herbage mass and pasture content of crude protein, neutral detergent fibre (NDF) and total FA were similar between HMF and LMF herds. Pasture offered to LMF herds had a higher concentration of monounsaturated fatty acids (MUFA). A strong negative relationship (*r* = -0.40) was evident between milk fat percentage and pasture crude protein content for MMF herds (3.31–3.94% milk fat).

**Conclusions:**

This research reports improved milk protein percentage, milk casein percentage and cheddar cheese yield from HMF herds compared to LMF herds. Milk processability was not impacted by low milk fat percentage. Pasture NDF and total fatty acid content was similar in HMF herds and LMF herds. Milk fat percentage had a strong negative association (*r* = -0.40) with pasture crude protein content in MMF herds (MF 3.31–3.94%). Correlation values between pasture chemical and FA composition and milk fat percentage in LMF herds and HMF herds were low, indicating that diet is not the only causative factor for variation in milk fat of grazing dairy cows. Comparison of milk fatty acid composition from herds with and without milk fat depression suggests that there may be other fatty acids apart from CLA t10, c12 that contribute to the inhibition of milk fat synthesis during milk fat depression in grazing herds.

## Background

Milk fat is the most variable component of bovine milk and maintaining adequate milk fat percentage continues to be a challenge on grazing dairy farms at key times of the year [1]. Although both milk fat percentage and composition are influenced by factors such as genetics, stage of lactation, season and cow health; the diet of the cow has a profound effect on milk fat production [2, 3]. The normal milk fat percentage for Holstein herds has been defined as between 3.4% and 4.0% with some seasonal variation [4]. The average milk fat percentage of the Irish dairy cow population is 4.22%, where the predominant breed used is Holstein Friesian and where the diet is predominantly pasture based [5]. A reduction in milk fat yield in the presence of normal or expected milk yield, or yield of other milk components including milk protein, can be identified as milk fat depression [6]. Often, this depression may be up to a 50% reduction in milk fat yield with a greater decline in the *de-novo* synthesised portion of milk fatty acids [7]. Identification of milk fat depression in herds has been based on bulk tank milk analysis where milk fat percentage is < 3.30% and milk protein percentage is a minimum of 3.20% [1]. The reduction in milk fat percentage or yield has largely been attributed to a shift in the biohydrogenation of unsaturated FA in the rumen, resulting in the production of milk fat inhibiting isomers of conjugated linoleic acid (CLA), such as CLA t10, c12. Low rumen pH which has been highlighted as a common issue of grazing dairy cows [8–10] is known to disrupt the rumen environment and result in altered microbial processes involved in the biohydrogenation of PUFA’s [11]. Low rumen pH in grazing cows has been associated with lower milk fat percentage [12]. However, the feeding conditions and interactions with rumen environment required for MFD are not fully understood. Milk fat depression in confinement milk production systems, is often the result of high levels of starch and low levels of NDF in the diet [13]. Meanwhile, the occurrence of high water intake, high unsaturated fatty acid (UFA) intake, intense production of volatile fatty acids (VFA) instead of lactic acid and selective feeding behaviour are thought to promote the condition in grazing scenarios [12]. Variability in grass composition due to season, weather and grassland management can further intensify challenges in maintaining milk fat levels [12, 14, 15].

Previous work has focused on investigating altered biohydrogenation pathways of UFA’s, C18:1c9 and C18:2c9,12 as a cause of MFD, through the use of oil supplements in total mixed ration (TMR) diets [16–18]. However, linolenic acid (LNA) which is the predominant FA in perennial ryegrass also has the ability to produce these milk fat inhibiting CLA isomers [19]. Details of the altered biohydrogenation of LNA, is limited. Likewise, the implications of MFD for milk processability and effects on other milk components are unknown. Investigation of the factors relating to pasture and milk composition that alter the milk fat percentage and FA composition of milk from grazing dairy cows will identify dietary risk factors in grazing diets that encourage the onset of MFD in cows. Therefore, the objective of this study was to compare the chemical and FA composition of pasture offered to, and milk produced from grazing herds with and without MFD.

## Results

Estimated feeding rate of concentrates for HMF, MMF and LMF herds was 2.3, 2.8 and 2.7 kg DM/cow/day respectively (Table 1).

**Table 1:** Estimated feeding rate and chemical composition of concentrate supplement offered to cows

Item	**HMF**^a^	**MMF**^a^	**LMF**^a^
Estimated feeding rate (kg DM/cow/day)	2.3	2.8	2.7
DM (%)	89.5	89.5	90.1
Chemical composition (% of DM)			
Crude protein	16.4	17.5	16.7
NDF	25.2	25.6	27.1
ADF	13.2	13.8	14.4
Ash	10.4	9.7	9.6
Starch	21.7	20.3	20.8
Oil—Ether extract	2.8	2.7	2.8

^a^HMF (> 3.95% milk fat) = High milk fat herds, MMF (3.31 – 3.94% milk fat) = Medium milk fat herds, LMF = Low milk fat herds (< 3.30% milk fat)

Milk fat percentage and cheddar cheese yield differed between all three herd classifications (HMF, MMF and LMF) (*P* < 0.001) (Table 2). Milk protein percentage and milk casein percentage of the HMF herds were higher than that of the LMF herds (*P* < 0.001). However, no differences were observed between HMF, MMF and LMF herds for MUN, milk lactose percentage and SCC. Likewise, milk processability parameters including milk pH, milk rennet coagulation time and milk ethanol stability were not different for the HMF, MMF and LMF herds.

**Table 2:** Mean milk composition and milk processability for HMF, MMF and LMF herds

Item	**HMF**^1^	**MMF**^1^	**LMF**^1^	**S.E.M**^**2**^
Milk Composition				
Milk fat (%)	4.18^a^	3.61^b^	3.08^c^	0.032
Milk protein (%)	3.66^a^	3.45^b^	3.43^b^	0.030
Milk lactose (%)	4.56	4.55	4.56	0.011
MUN (mg/dL)	20.77	21.80	21.77	0.140
Milk casein (%)	2.91^a^	2.72^b^	2.70^b^	0.027
SCC (‘000 cells)	106	133	105	19.0
Cheddar cheese yield (kg/100 kg milk)	10.69^a^	9.56^b^	8.76^c^	0.066
Milk Processability				
Milk pH	6.69	6.6	6.65	0.022
Milk rennet coagulation time (min)	2.80	3.40	2.90	0.256
Milk Ethanol Stability (%)	84.8	82.4	82.8	1.33

^1^HMF (> 3.95% milk fat) = High milk fat herds, MMF (3.31 – 3.94% milk fat) = Medium milk fat herds, LMF = Low milk fat herds (< 3.30% milk fat)

^2^S.E.M = Standard error of the mean

^a,b,c,^ Means in the same row with different superscripts differ (*P* < 0.05)

Milk from LMF herds had lower concentrations of C10:0, C12:0, total saturated FA and a lower ratio of omega-3 to omega-6 FA compared to HMF herds (Table 3). However, concentrations of C18:1c9, C18:2 *trans* isomers, C18:2c9,12, C20:1c11, C21:0, C20:3c8,11,14, C20:4c5,8,11,14 total MUFA, total PUFA, total omega-6 and total omega-9 and ratio of omega-6 to omega-3 FA were higher in milk from LMF herds.

**Table 3:** Mean milk FA composition of HMF, MMF and LMF herds (g/100 g FA)

	**HMF**^1^	**MMF**^1^	**LMF**^1^	**S.E.M**^**2**^
FA (g/100 g FA)				
C4:0	1.68	1.70	1.66	0.081
C6:0	1.55^a^	1.38^b^	1.45^a,b^	0.048
C8:0	1.26	1.21	1.20	0.021
C10:0	3.37^a^	3.24^a,b^	3.16^b^	0.068
C11:0	0.11	0.10	0.10	0.007
C12:0	4.36^a^	4.15^a,b^	4.05^b^	0.099
C13:0	0.27	0.25	0.25	0.010
C14:0	12.86	12.88	12.57	0.184
C14:1c9	1.03^a^	1.12^b^	1.11^a,b^	0.031
C15:0	1.48	1.45	1.43	0.032
^3^ Total *de-novo* FA	27.76	27.49	26.94	0.427
C16:0	29.67	29.78	28.95	0.362
C16:1c9	1.43	1.43	1.37	0.033
C17:0	0.81	0.81	0.82	0.012
C18:0	11.53	11.10	11.28	0.250
C18:1t9	0.39^a^	0.46^a,b^	0.50^b^	0.027
C18:1t11	3.25	3.11	3.27	0.148
C18:1c9	20.08	20.90	21.38	0.384
C18:1c11	0.45	0.46	0.48	0.015
C18:2c 9,12	1.11^a^	1.25^b^	1.34^c^	0.037
C18:3c9,12,15	0.74	0.72	0.76	0.017
C20:0	0.14	0.14	0.14	0.005
C20:5c 5, 8, 11, 14, 17	0.09	0.09	0.10	0.002
CLA c9, t11	1.43	1.46	1.56	0.073
CLA t10, c12	0.06	0.05	0.05	0.003
Total CLA	1.48	1.52	1.64	0.074
Total Saturated FA	69.42^a^	68.36^a,b^	67.36^b^	0.526
Total MUFA	23.08^a^	23.97^a,b^	24.37^b^	0.392
Total PUFA	2.36^a^	2.54^b^	2.69^c^	0.055
Total Unsaturated FA	25.45^a^	26.45^ab^	26.97^b^	0.416
Total Omega 3 FA	1.00	0.96	1.01	0.019
Total Omega 6 FA	1.39^a^	1.56^b^	1.67^b^	0.042
Total Omega 7 FA	1.92	1.89	1.83	0.036
Total Omega 9 FA	20.14^a^	20.96^a,b^	21.45^b^	0.384
Ratio Omega 3:Omega 6	0.73^a^	0.63^b^	0.62^b^	0.018
Ratio Omega 6:Omega 3	1.41^a^	1.64^b^	1.68^b^	0.048

^1^HMF (> 3.95% milk fat) = High milk fat herds, MMF (3.31 – 3.94% milk fat) = Medium milk fat herds, LMF = Low milk fat herds (< 3.30% milk fat)

^2^S.E.M = Standard error of the mean

^a,b,c,^ Means in the same row with different superscripts differ (*P* < 0.05)

^3^Total de-novo FA = sum of FA from C4 to C15:0 inclusive

No difference in pre-grazing herbage mass, DM %, NDF, ADF, ash and ether extract was observed for HMF, MMF and LMF herds (Table 4). However, pasture offered to cows from HMF herds tended to be higher in WSC than LMF herds. Pasture total FA content was not different between HMF, MMF and LMF herds (*P* > 0.05). Short chain FA composition of pasture was similar for both HMF, MMF and LMF herds. Pasture content of C15:0 tended to be higher for LMF herds (*P* < 0.10). Pasture content of C16:0 was higher for MMF and LMF herds compared to HMF herds (*P* < 0.05). No differences were observed in pasture content of the C18:2c9,12 and C18:3c9,12,15; the main FA’s found in pasture (*P* > 0.05). Total saturated FA and MUFA content of pasture was higher for LMF compared to HMF herds. Total PUFA content of pasture was higher for HMF compared to LMF herds.

**Table 4:** Mean pasture chemical composition and FA composition of HMF, MMF and LMF herds

Item	**HMF**^1^	**MMF**^1^	**LMF**^1^	**S.E.M**^**2**^
Pre-grazing herbage mass (kg/DM ha)	1821	2012	1660	124.0
DM (%)	17.41	17.56	17.29	0.492
Chemical composition (% of DM)				
Crude protein	17.66	18.22	18.33	0.756
NDF	43.40	44.93	45.25	0.782
ADF	19.84	20.89	20.98	0.465
Ash	8.37	8.41	8.60	0.232
WSC	5.65	4.78	4.01	0.571
Oil—Ether extract	3.03	2.91	2.74	0.135
FA (mg/g DM)				
Total FA (mg/g DM)	28.25	26.54	25.46	1.233
FA (g/kg of total FA)				
C4:0	0.11	0.12	0.12	0.011
C6:0	0.19^a^	0.22^a,b^	0.27^b^	0.025
C8:0	0.15^a^	0.22^b^	0.22	0.025
C12:0	0.28	0.34	0.36	0.032
C14:0	0.63	0.66	0.75	0.041
C15:0	0.17	0.20	0.21	0.014
C16:0	18.64^a^	20.21^a,b^	20.89^b^	0.685
C16:1c9	0.22	0.25	0.28	0.020
C17:0	0.22^a^	0.24^a,b^	0.26^b^	0.015
C18:0	1.66^a^	1.82^a,b^	2.01^b^	0.096
C18:2c 9,12	14.80	15.23	14.78	0.349
C18:3c6,9,12	0.10	0.12	0.15	0.021
C18:3c9,12,15	57.81	54.65	53.72	1.400
C20:0	0.38	0.40	0.42	0.027
C21:0	0.16	0.19	0.20	0.025
C22:0	0.63	0.65	0.71	0.030
C24:0	0.44	0.42	0.49	0.024
C24:1c15	0.13	0.15	0.16	0.008
Total Saturated FA	23.96^a^	26.19^a,b^	27.55^b^	1.017
Total MUFA	3.00^a^	3.29^a,b^	3.65^b^	0.201
Total PUFA	72.72^a^	70.41^a,b^	68.71^b^	1.195
Total Unsaturated FA	75.81^a^	73.86^a,b^	72.30^b^	1.143
Total Omega 3 FA	57.91	54.75	53.81	1.399
Total Omega 6 FA	14.99	15.45	15.01	0.349
Total Omega 7 FA	0.68	0.71	0.75	0.045
Total Omega 9 FA	2.36^a^	2.56^a,b^	2.86^b^	0.158
Ratio Omega 3:Omega 6	3.99	3.64	3.68	0.163
Ratio Omega 6:Omega 3	0.27	0.28	0.28	0.012

^1^HMF (> 3.95% milk fat) = High milk fat herds, MMF (3.31 – 3.94% milk fat) = Medium milk fat herds, LMF = Low milk fat herds (< 3.30% milk fat)

^2^S.E.M = Standard error of the mean

^a,b,c,^ Means in the same row with different superscripts differ (*P* < 0.05)

Analysis of the Entire group (*n* = 94) revealed a positive correlation (*r* = 0.22) between milk fat percentage and pasture WSC content (*P* < 0.05) (Table 5). Milk fat percentage tended to be negatively correlated with pasture NDF content (*r* = -0.20) and positively correlated with pasture ether extract (*r* = 0.19) across the Entire group (*n* = 94). When the data set was split into categories based on milk fat percentage, no relationship was found between milk fat percentage and any of the pasture composition parameters including crude protein, NDF, ADF, WSC and ether extract for the HMF (MF < 3.95%) and LMF (MF > 3.30%) herds. However, a strong negative relationship (*r* = -0.40) was found between milk fat percentage and pasture crude protein content in the MMF herds (MF 3.31–3.94%). Milk fat percentage was not correlated with pasture total FA content or pasture content of C18:2c9,12, total saturated FA, total MUFA, total PUFA or omega-3 FA for the Entire group and for the HMF and LMF herds. A weak negative correlation existed between pasture C18:3c9,12,15 content and milk fat content for the Entire group (*r* = -0.14) (*P* = 0.17). Milk fat percentage tended to be positively correlated with pasture total saturated FA (*P* < 0.1) and negatively correlated with pasture omega-3 FA (*P* < 0.1) for the MMF herds.

**Table 5:** Correlation estimates of milk fat percentage on pasture chemical composition and FA composition

		Correlation Estimates		
Item	**Entire**	**HMF**^1^	**MMF**^1^	**LMF**^1^
	(*n* = 94)	(*n* = 24)	(*n* = 42)	(*n* = 28)
Chemical composition (% of DM)				
Crude protein	-0.17	-0.10	-0.40**	-0.26
NDF	-0.20 †	-0.23	-0.09	-0.14
ADF	-0.12	-0.11	0.19	0.08
WSC	0.22 *	0.32	0.12	-0.07
Oil—Ether extract	0.19 †	0.06	0.12	0.03
Total FA (mg/g DM)	0.07	-0.10	-0.25	-0.13
FA (g/100 g FA)				
C18:2c9,12	0.01	-0.25	0.25	-0.06
C18:3c9,12,15	-0.14	-0.08	-0.06	0.03
Total Saturated FA	-0.17	-0.06	0.27 †	0.05
Total MUFA	-0.17	-0.13	0.17	0.11
Total PUFA	0.16	0.05	-0.26	-0.06
Total Omega 3 FA	0.14	0.11	-0.28 †	-0.04
Total Omega 6 FA	0.01	-0.26	0.26 †	-0.06
Total Omega 7 FA	-0.08	-0.02	0.20	0.02
Total Omega 9 FA	-0.16	-0.11	0.19	0.12
Ratio Omega 3:Omega 6	0.09	0.24	-0.34 *	0.01
Ratio Omega 6:Omega 3	-0.14	-0.37 †	0.45 **	-0.03

^1^HMF (> 3.95% milk fat) = High milk fat herds, MMF (3.31 – 3.94% milk fat) = Medium milk fat herds, LMF = Low milk fat herds (< 3.30% milk fat)

^†^*P* < 0.10; * *P* < 0.05; ** *P* < 0.01; *** *P* < 0.001

Milk fat total CLA concentration was not correlated with pasture total FA content or pasture content of C18:2c 9,12, C18:3c 9,12,15, saturated FA and PUFA for the Entire group of herds (Table 6). Milk fat total CLA concentration tended to be positively correlated with pasture crude protein content (*r* = 0.40) and total FA content (*r* = 0.36) for the MMF herds.

**Table 6:** Correlation estimates of milk total CLA concentration with pasture composition and FA composition for the Entire, HMF, LMF and MMF herd categories

		Correlation Estimates		
Item	**Entire**	**HMF**^1^	**MMF**^1^	**LMF**^1^
	(*n* = 92)	(*n* = 24)	(*n* = 41)	(*n* = 27)
Chemical composition (% of DM)				
Crude protein	0.17	-0.05	0.40†	-0.22
NDF	0.09	-0.29	0.05	0.06
ADF	-0.01	-0.40†	-0.12	0.08
WSC	-0.16	0.04	-0.07	0.29
Oil—Ether extract	-0.03	-0.2	0.02	0.11
Total FA (mg/g DM)	0.1	0.08	0.36 †	0.12
FA (g/100 g FA)				
C18:2c9,12	-0.13	-0.02	-0.23	0.04
C18:3c9,12,15	0.15	-0.24	0.04	0.16
Total Saturated FA	-0.03	-0.17	-0.21	-0.24
Total MUFA	-0.1	-0.39 †	-0.12	-0.27
Total PUFA	0.04	0.22	0.2	0.25
Total Omega 3 FA	0.066	0.19	0.22	0.21
Total Omega 6 FA	-0.12	-0.03	-0.24	0.05
Total Omega 7 FA	-0.04	-0.33	-0.03	-0.21
Total Omega 9 FA	-0.13	-0.39 †	-0.15	-0.27
Ratio Omega 3:Omega 6	0.11	0.09	0.25	0.16
Ratio Omega 6:Omega 3	-0.07	-0.06	-0.32	-0.07

^1^HMF (> 3.95% milk fat) = High milk fat herds, MMF (3.31 – 3.94% milk fat) = Medium milk fat herds, LMF = Low milk fat herds (< 3.30% milk fat)

^†^*P* < 0.10; * *P* < 0.05; ** *P* < 0.01; *** *P* < 0.001

A positive correlation was observed between milk fat total omega-3 FA concentration and pasture total FA content for the Entire group (*r* = 0.22) and for the MMF herds (*r* = 0.36) (*P* < 0.05) (Table 7). Likewise, milk fat total omega-3 FA was positively correlated with pasture crude protein content for the MMF herds (*r* = 0.40) (*P* < 0.05). Pasture PUFA content tended to positively increase concentrations of total omega-3 FA in milk across the Entire group (*r* = 0.20) (*P* < 0.1).

**Table 7:** Correlation estimates of milk total omega-3 FA concentration with pasture chemical composition and FA composition for the Entire, HMF, LMF and MMF herd categories

		Correlation Estimates		
Item	**Entire**	**HMF**^1^	**MMF**^1^	**LMF**^1^
	(*n* = 94)	(*n* = 24)	(*n* = 42)	(*n* = 28)
Chemical composition (% of DM)				
Crude protein	0.12	-0.05	0.40*	-0.22
NDF	-0.05	-0.29	0.05	0.06
ADF	-0.15	-0.40†	-0.12	0.08
WSC	0.04	0.04	-0.07	0.29
Oil—Ether extract	-0.02	-0.2	0.02	0.11
Total FA (mg/g DM)	0.22*	0.08	0.36*	0.12
FA (g/100 g FA)				
C18:2c9,12	-0.12	-0.02	-0.23	0.04
C18:3c9,12,15	0.02	-0.24	0.04	0.16
Total Saturated FA	-0.19†	-0.17	-0.21	-0.24
Total MUFA	-0.20†	-0.39†	-0.12	-0.27
Total PUFA	0.20†	0.22	0.2	0.25
Total Omega 3 FA	0.20†	0.19	0.23	0.21
Total Omega 6 FA	-0.12	-0.03	-0.24	0.05
Total Omega 7 FA	-0.15	-0.33	-0.03	-0.21
Total Omega 9 FA	-0.21*	-0.39†	-0.15	-0.27
Ratio Omega 3:Omega 6	0.19†	0.09	0.25	0.16
Ratio Omega 6:Omega 3	-0.18†	-0.06	-0.32*	-0.07

^1^HMF (> 3.95% milk fat) = High milk fat herds, MMF (3.31 – 3.94% milk fat) = Medium milk fat herds, LMF = Low milk fat herds (< 3.30% milk fat)

^†^*P* < 0.10; * *P* < 0.05; ** *P* < 0.01; *** *P* < 0.001

A strong positive correlation was observed between milk fat percentage and milk protein percentage (*r* = 0.42) (*P* < 0.001), milk casein percentage (*r* = 0.41) (*P* < 0.001) and cheddar cheese yield (*r* = 0.94) (*P* < 0.001) for the Entire group (Table 8). The relationship between milk fat percentage and milk C18:0, the final product of C18:2c9,12 and C18:3c9,12,15 biohydrogenation, was negative for the HMF herds (*r* = -0.11) whereas this was positive for the LMF herds (*r* = 0.13). A strong negative relationship was observed between milk fat percentage and milk C18:1c9 for the HMF herds (*r* = -0.43) (*P* < 0.05) whereas the inverse of this relationship was observed in the LMF herds (*r* = 0.43) (*P* < 0.05). A moderate to strong negative relationship with milk fat percentage was observed in all of the milk omega-6 FA (C18:2c9,12; C18:3c6,9,12; C20:2c11,14; C20:3c8,11,14; C20:4c5,8,11,14 and C22:2c13,16) for the HMF herds while this relationship was generally positively weak to moderate for the majority of these milk omega-6 FA in the LMF herds. A strong negative correlation between milk fat percentage and milk C18:2c9,12 was observed in the Entire group (*r* = -0.41) (*P* < 0.001). Milk fat percentage and milk C18:3c9,12,15 were negatively correlated for the MMF herds (*r* = -0.34) (*P* < 0.05). No relationship was found between milk fat percentage and the two isomers of C18:2; CLA c9,t11 and CLA t10,c12, for the Entire group. Milk fat percentage and milk total saturated FA were positively correlated for the Entire group (*r* = 0.28) (*P* < 0.01). The opposite trend was observed for the relationships between milk fat percentage with milk total MUFA (*r* = -0.24) (*P* < 0.05) and total PUFA content (*r* = -0.39) (*P* < 0.001) whereby milk total MUFA and total PUFA content decreased as milk fat percentage increased within the Entire group. Similarly, milk fat percentage and milk total PUFA were negatively correlated for the Entire group. A moderate negative relationship (*r* = -0.36) (*P* < 0.05) was observed between milk fat percentage and milk omega-3 FA for MMF herds. Although not significant, a weak positive relationship was observed between milk fat percentage and milk total *de-novo* synthesized FA for the Entire group (*r* = 0.10).

**Table 8:** Correlation estimates of milk fat percentage on milk composition and milk FA composition

		Correlation Estimates		
Item	**Entire**	**HMF**^1^	**MMF**^1^	**LMF**^1^
	(*n* = 94)	(*n* = 24)	(*n* = 42)	(*n* = 28)
Milk Composition				
Milk protein	0.42***	0.23	0.12	-0.20
Milk lactose	0.01	0.06	-0.07	-0.21
Milk SCC	0.02	-0.06	-0.05	0.23
Milk casein	0.41***	0.19	0.10	-0.25
MUN	-0.15	-0.28	-0.28†	-0.24
Cheddar cheese yield	0.94***	0.67***	0.78***	0.73***
Milk FA (g/kg of total FA)				
C4:0	-0.02	0.12	-0.01	-0.31
C6:0	0.10	0.28	-0.08	-0.21
C8:0	0.18†	0.32	0.06	-0.29
C10:0	0.16	0.14	0.06	-0.41*
C11:0	0.16	0.28	-0.11	-0.45*
C12:0	0.18	0.19	0.03	-0.33†
C13:0	0.03	0.12	-0.11	-0.37†
C14:0	0.10	0.13	0.12	-0.33†
C14 1c9	-0.20†	-0.2	-0.18	0.02
C15:0	0.05	0.17	-0.08	-0.40
C16:0	0.26*	0.42*	0.31*	-0.24
C16 1c9	0.16	-0.14	0.13	-0.20
C17:0	-0.09	0.05	-0.06	-0.40*
C18:0	-0.01	-0.11	0.05	0.13
C18 1t9	-0.32**	-0.25	-0.38*	0.04
C18 1t11	0.003	-0.08	-0.16	0.33 †
C18 1c9	-0.24*	-0.43*	-0.17	0.43*
C18 1c11	-0.13	-0.46*	-0.13	0.27
C18 2t	-0.35***	-0.29	-0.16	-0.34†
C18:2c 9, 12	-0.41***	-0.43*	-0.21	0.33†
C18:3c 6,9,12	-0.22*	-0.29	-0.25	0.23
C18:3c 9,12,15	-0.13	-0.15	-0.34*	0.13
CLA c9, t11	-0.13	-0.15	-0.21	0.22
CLA t10, c12	0.06	0.07	-0.12	0.30
C20:0	-0.16	-0.01	0.11	0.04
C20:1c 11	-0.30**	-0.29	0.01	0.10
C20:2c 11,14	-0.15	-0.51*	-0.24	0.38*
C20:3c 8,11,14	-0.49****	-0.46*	-0.35*	-0.01
C20:3c 11,14,17	0.18†	-0.01	-0.07	0.58**
C20:4c 5,8,11,14	-0.44***	-0.49*	-0.34*	0.16
C20:5c 5,8,11,14,17	0.01	-0.07	0.13	-0.12
C21:0	-0.35***	-0.09	-0.06	0.12
C22:0	0.03	-0.28	0.15	0.26
C22:1c 13	0.10	0.15	0.04	0.28
C22:2c 13,16	-0.11	-0.29	-0.31*	-0.04
C22:5c 7,10,13,16,19	-0.21*	-0.50*	-0.26	0.02
C22:6c 4,7,10,13,16,19	0.14	0.04	-0.22	0.24
C23:0	-0.04	-0.28	0.06	0.13
C24:0	-0.07	-0.21	0.24	0.24
C24:1c 15	0.16	0.07	-0.18	0.20
Total Saturated FA	0.28**	0.46*	0.26†	-0.40*
Total MUFA	-0.24*	-0.47*	-0.17	0.40*
Total PUFA	-0.39***	-0.48*	-0.28†	0.39*
Total Omega-3 FA	-0.11	-0.22	-0.36*	0.05
Total Omega-6 FA	-0.43***	-0.49*	-0.25	0.35†
Total Omega-7 FA	0.10	-0.31	0.05	-0.08
Total Omega-9 FA	-0.24*	-0.43*	-0.17	0.43*
Ratio Omega-3: Omega-6	0.41***	0.36†	0.14	-0.06
Ratio Omega-6: Omega-3	-0.38***	-0.32	-0.18	0.08
Total CLA	-0.13	-0.14	-0.21	0.31
^2^ Total *de-novo* FA	0.10	0.13	0.04	-0.41*

^1^HMF (> 3.95% milk fat) = High milk fat herds, MMF (3.31 – 3.94% milk fat) = Medium milk fat herds, LMF = Low milk fat herds (< 3.30% milk fat)

^2^Total de-novo FA = sum of FA from C4 to C15:0 inclusive

^†^*P* < 0.10; * *P* < 0.05; ** *P* < 0.01; *** *P* < 0.001

## Discussion

### Differences in milk composition due to herd milk fat category

The relationships between pasture and milk components of Irish grazing dairy farms were examined to determine if milk fat percentage was related to pasture composition, milk processability, milk composition or milk FA composition. It is generally accepted that MFD in lactating dairy cows is caused by feeding diets high in starch and low in fibre or by feeding diets high in UFA [7, 20, 21]. Furthermore, investigations into the causes of MFD have mainly focused on animals fed TMR diets [22]. However, little is known about the relationship between pasture composition and MFD in grazing scenarios [1].

Milk payment schemes in Ireland and many other countries are calculated based on kilograms of fat and protein supplied, with a volume charge, subtracted for each litre of milk supplied [23]. Hence, improved milk composition for HMF herds in comparison to LMF herds translates to an economic benefit to milk suppliers. Some studies have demonstrated no effect of MFD on milk protein percentage in cows fed a MFD inducing diet (milk fat% = 2.59) and cows fed a control diet (milk fat% = 3.46) [24]. Whereas other studies have reported a higher milk protein percentage of 3.60% in cows with MFD. Different relationships existed between milk protein percentage and milk fat percentage in HMF herds compared to LMF herds. This may be attributable to the higher WSC content of pasture offered to HMF herds. An increase in energy supply owing to the increased pasture WSC content could optimise rumen microbial protein synthesis and thus milk protein synthesis for HMF herds [25]. Although not significant, the results of the present study suggest a negative relationship exists between milk fat percentage and milk protein percentage in LMF herds, which agrees with other studies where milk fat decreased as milk protein increased in cows with MFD [7]. It is also possible that the inverse association between milk fat percentage and milk protein percentage in HMF herds and LMF herds may be related to the definition of MFD [1] used to categorize herds into their groups as all herds included the study were required to have a milk protein percentage of > 3.20% according to the definition of MFD [1].

Undesirable milk processability characteristics can limit the production of milk products, such as butter or cheese for milk processors [26]. Milk processability parameters such as those reported in the current study have not previously been investigated in herds with and without MFD. Our results for milk processability characteristics are similar to other studies for pasture fed cows [26, 27] and demonstrated that milk fat percentage had no effect on processability characteristics including milk pH, milk RCT and milk ethanol stability.

Concentrations of C10:0 and C12:0 FA, which account for a significant proportion of the *de-novo* synthesized FA, were significantly higher in the HMF herds compared to the LMF herds. Considering the *de-novo* synthesized FA are lower in cows with MFD [28], the lower concentration of *de-novo* FA in milk from LMF herds, may suggest that milk fat synthesis was negatively affected by conditions that alter the rumen environment, such as low rumen pH [12], which is common in grazing scenarios [8].

Low rumen pH has been proposed to induce MFD through alterations in rumen fermentation [13]. Previous studies have highlighted the potential for low rumen pH in grazing dairy cows [29] with approximately 50% exhibiting rumen pH below < 5.8 [8]. Lower rumen pH has been associated with lower milk fat percentage [9], lower milk fat: protein ratio and lower acetate to propionate ratio [30] and reductions in milk fat yield [10]. Furthermore, where rumen pH was only mildly suboptimal (pH 5.8), a 31% reduction in the extent of C18:2c9,12 biohydrogenation was associated with a 35% reduction in NDF disappearance [31].

When the rumen environment is altered, FA including C18:2c9,12 and C18:3c9,12,15 undergo an altered pathway of biohydrogenation and result in the creation of CLA t10, c12 isomers instead of CLA c9, t11 isomers [32]. Many studies have attributed the onset of MFD in dairy cows to the presence of the milk fat inhibiting isomer CLA t10, c12 [33–35]. Despite fitting the description of MFD [1], milk from LMF herds did not display a higher concentration of CLA t10, c12 compared to milk from HMF herds. Additionally, the correlation between milk fat percentage and CLA t10, c12 for the Entire group was weak and not significant. In agreement with results of this study, many authors have indicated that an increase in ruminal CLA t10, c12 formation does not provide a universal explanation for the reduction in milk fat production during diet-induced MFD and have suggested that other biohydrogenation intermediates of C18:2c9,12 and C18:3c9,12,15 may also be involved [32, 35]. For example, average reductions in milk fat percentage and milk fat yield by 52% and 55%, respectively were demonstrated through the use of CLA isomers CLA c8, t10; CLA c9, t11; CLA c10, t12 and CLA c11, t13 [36]. Reductions in milk fat yield by 15% were also attributed to the isomer CLA t9, c11 [34]. Likewise, reductions in milk fat percentage and milk fat yield by 23% and 21%, respectively were demonstrated using an abomasal infusion containing the CLA c10, t12 isomer [37]. Despite this, the only two CLA isomers measured in this study CLA c9, t11 and CLA t10, c12, were not reduced in milk from LMF herds compared to HMF herds. Nor was there any correlation for these isomers and milk fat percentage for the Entire group. It is possible that other CLA isomers linked to MFD such as those discussed above, may have impacted milk fat synthesis in this study. However, no measurements for other CLA isomers were available for our study.

### Pasture composition: differences with herd milk fat category

Reductions in milk fat production have previously been associated with increased dietary intake of C18:2c9,12 and C18:3c9,12,15 in dairy cows fed grass based diets [38]. Given that long chain FA are preformed and originate from the diet [39], the absence of a difference in pasture concentration in the majority of these FA suggests that differences in milk concentration of these FA may have resulted from a difference in the extent of biohydrogenations in the rumen. Similar to previous studies where cows experienced MFD on a grass silage diet supplemented with sunflower oil [40], the LMF herds also displayed lower concentrations of saturated FA and higher concentrations of MUFA in milk compared with the HMF herds. On account of an increased omega 3 to omega 6 ratio, the FA composition of milk from the LMF herds may be more desirable in reducing the risk of many of the highly prevalent chronic diseases of modern society such as cardiovascular disease, cancer, and inflammatory and autoimmune diseases. Whereas increased levels of omega-3 PUFA (a low omega-3 to omega-6 ratio) exert suppressive effects on these diseases [41].

Given that numerous studies have attributed changes in milk fat percentage to diet composition [18, 40, 42], it was surprising that pasture offered to both HMF and LMF herds was similar in composition, yet cows displayed very different milk fat percentages. Nevertheless, it must be noted that the pasture sampled may not have been identical in composition to the pasture that caused the reduction in milk fat percentage. Dietary induced MFD typically takes up to 14 days before a reduction in milk fat percentage is observed [17, 18, 21]. Hence, the pasture fed during the 14 days previous to sampling was likely to have caused the MFD. One limitation of this study is that pasture was sampled subsequent to herd selection, however it was not possible to sample the pasture before the farms of interest were identified. Perhaps further research could consider the relationship between milk fat percentage and pasture consumed prior to MFD occurrence.

Pre-grazing herbage mass (above 4 cm) of pasture offered to the HMF herds was similar to the pasture offered to the LMF herds (1821 vs 1660 kg DM/ha). Evidence of the effects of pre-grazing herbage mass on milk fat percentage is conflicting. Some studies have attributed lower milk fat percentages in grazing scenarios to inadequate fibre intake from lower pre-grazing herbage mass [1, 8, 12]. Whereas other studies have reported no effect of pre-grazing herbage mass of perennial ryegrass [43–45].It is possible that the effect of pre-grazing herbage mass on milk fat percentage in dairy cows may be different depending on season as NDF content of perennial ryegrass increases throughout the growing season regardless of pre-grazing herbage mass [46]. The absence of a difference in dietary fibre content of pasture in the current study was not expected as NDF has been found to have a direct link with milk fat synthesis [17, 18]. Acetate, which is a VFA produced from the fermentation of fibrous feeds in the rumen contributes to 50% of milk fat production [47]. Hence, a higher dietary fibre content was expected in pasture offered to the HMF herds as a result of the increased milk fat production.

A higher crude protein content was expected to explain the lower milk fat percentage of cows in the LMF herds as high nitrogen intake is associated with increased FA content in grass which is a contributing factor of MFD [48, 49]. In the current study, a weak negative correlation was observed between pasture crude protein and milk fat percentage for the Entire group (*r* = -0.17), which agrees with the findings of these studies. A stronger negative relationship was observed for the MMF herds compared to LMF herds. Given that only 10–20% of herds have milk fat percentages below 3.30% during the grazing season [1], the correlation for the MMF herds (milk fat percentage 3.31—3.94%) is perhaps more relevant, as it is applicable to a relatively larger number of herds.

The moderate negative correlation observed in this study between milk fat percentage and pasture PUFA content for the MMF group supports the theory that intake of PUFA, which are toxic to rumen microbes, negatively impacts rumen fermentation and hence milk fat production [12]. The average total FA content of pasture offered to HMF herds and LMF herds was quite low (27.4 mg/g and 26.1 mg/g respectively) and more comparable with total FA contents of diets where cows recovered from MFD rather than induced MFD. Reductions in milk fat yield by 30% were reported in dairy cows offered a diet containing 5.8% total FA with recovery achieved at a dietary concentration of 4.2% total FA [18]. Similarly, MFD was induced in cows at a dietary concentration of 5.5% total FA, whereas recovery from MFD was achieved by feeding a diet with a dietary concentration of 2.6% total FA [17]. The slightly smaller range in pasture total FA for HMF herds (13.3 to 38.9 mg/g) (S.D = 6.05) in comparison to LMF herds (13.5 to 43.5 mg/g) (S.D = 7.62) may have been beneficial in terms of improving biohydrogenation and rumen fermentation. Comparison with these studies suggest that total FA concentration of pasture in the present study may not have been high enough to negatively affect rumen microbes and cause UFA’s to be metabolised through the altered pathway of biohydrogenation. It is also, possible that pasture intake although not measured in this study, may have varied greatly between herds, resulting in very different total FA intake between herds.

Production of the CLA isomers; CLA c9,t11 and CLA t10, c12, both which have been linked with causing MFD [12], results mainly from the biohydrogenation of C18:2c9,12 and C18:3c9,12,15. Considering the combination of both C18:2c9,12 and C18:3c9,12,15 contributed approximately 70% of the total FA content of the pasture offered to both groups, it could be expected that these isomers would exert a significant impact on the production of milk fat. However, C18:2c9,12 and C18:3c9,12,15 content of pasture offered to both groups were similar in this study. This may suggest that concentration of these FA in pasture were not associated with the reduction in milk fat percentage, again however pasture intake and hence FA intake were not determined.

Although C18:2c9,12 is more potent than C18:1c9 for depressing milk fat [16, 50] where rumen pH is suboptimal, C18:1c9 can also alter the biohydrogenation pathways to favour the production of CLA t10, c12 and related intermediates from UFA already in the diet, resulting in milk fat depression [16]. The lower MUFA content of pasture offered to LMF herds in comparison to HMF herds may explain the reduction in milk fat percentage in this study. This result is also supported by the negative correlation found between milk fat percentage and pasture MUFA content for the Entire group of herds. Contrary to this, some authors reported no difference in milk fat percentage or milk fat yield when diets supplemented with either MUFA or PUFA were fed [51, 52]. The similar pasture composition across groups suggests that other factors, such as cow genetics [1, 53] or possible variation in pasture intake [12] may have contributed to the difference in milk fat percentage between the HMF and LMF herds. Previous research has demonstrated a higher prevalence of MFD in cows with a high sub-index for milk yield while cows with a high sub-index for milk fat kg had a lower prevalence of MFD [1]. Similarly, MFD was reported to have a heritability of 5% in dairy cows [53].

### Relationships between milk fat percentage with pasture and milk fatty acid composition

Considering that fibre fermentation is responsible for 50% of milk fat production [47], the negative relationship between milk fat percentage and pasture NDF content, was not expected. Many authors have cited work reporting negative effects of a low fibre diet on milk fat production [17, 18, 54, 55]. However, in many cases where MFD was induced by feeding low fibre diets, a reduction in milk fat percentage or milk fat yield was not achieved without the presence of high dietary oil concentration [42], suggesting that altered biohydrogenation of UFA does not occur from reduced NDF content alone. Additionally, some authors have questioned the severity of the impact of low NDF in grazing diets, suggesting that the effect may be different to traditional maize based TMR type diets [12]. A reason for this may be that milk fat is more sensitive to rumen pH rather than dietary NDF concentration [54]. Also, durations of suboptimal rumen pH in cows fed pasture diets may not be as long in duration as for TMR type diets due to variation in feeding behaviour [56]. The negative relationship between milk fat percentage and pasture total FA content agrees with other studies where negative effects of FA load on rumen milk fat synthesis due to alterations in rumen biohydrogenation have been reported. However, the small range and low concentration of total FA in pasture offered to both HMF and LMF herds may explain why this relationship was weak. This low range may have occurred due to very similar grassland management protocols and genetically similar grasses now used by many grazing farmers in Ireland. The negative relationship between milk fat percentage and pasture content of C18:3c9,12,15 for the Entire group agrees with other studies where high dietary C18:3c9,12,15 concentration induced milk fat depression [16, 57, 58].

The current study was conducted from single-sampling of a relatively small number of herds in Ireland. Therefore, further research carried out over an extended period of time and consisting of a combined evaluation of cow genetics and dietary characteristics is warranted to further investigate factors related to variation in milk fat percentage in grazing dairy cows.

## Conclusion

Of the pasture characteristics examined, crude protein content had the highest correlation with milk fat percentage for the overall group having a negative relationship with milk fat percentage. Of the milk FA examined, concentrations of C10:0, C12:0, total saturated FA and ratio of omega-3 to omega-6 FA were lower in milk from LMF herds. Furthermore, a strong negative relationship was observed between milk fat percentage and concentration of C18:2c9,12 in milk for the Entire group. Pasture concentration of C18:3c9,12,15 was negatively correlated with milk fat percentage for the Entire group. Cheddar cheese yield, milk protein percentage and milk casein percentage were lower for LMF herds. Milk processability parameters were not different between HMF, MMF and LMF herds. Further research should consider cow genetics, characteristics of the pasture consumed prior to the milk fat percentage outcome and further refinement of fatty acids in milk.

## Methods

The study took the form of once-off, on-farm investigations (*n* = 94 dairy herds) gathering data on milk, grass and concentrate composition, together with an assessment of pasture and dietary management. Data was gathered by trained milk processor staff and treated anonymously thereafter, with herds identified by sample number only. These herds were located in the east and southeast of Ireland and were sampled on one occasion over 2 years. Sampling of farms was carried out in years 2018 (*n* = 35) and 2019 (*n* = 59) and during the months of May and June as the prevalence of MFD is high during this time of the year in Ireland (**1**). Herds used for the analysis were predominantly Spring calving, pasture-fed, Holstein–Friesian dairy herds. To be included in the study, herds had to be managed on a flat rate feeding system of between 1 and 4 kg of supplementary concentrate per cow per day.

In the week prior to sampling, eligible herds were selected based on MIRS analysis of the previous three bulk tank milk samples collected at an interval of approximately three days each as collected by the milk processor. To ensure a wide range of milk samples were available for analysis, both low milk fat percentage and high milk fat percentage herds were included in the sample. Milk sampling was carried out for herds suspected to have MFD i.e., herds with previous three bulk tank milk samples at 3.30% or below for milk fat percentage were sampled. In addition to this, for each MFD herd, sampling of a nearby herd (within 10 km) with a high milk fat percentage i.e., herds with previous three bulk tank milk samples at 3.95% or above for milk fat percentage, was carried out to ensure geographical area was not a confounding factor in the analysis and also to ensure milk samples with a good range in milk fat percentage were available for the study. As an inclusion criterion, all herds selected for the study had to have a normal bulk tank milk protein percentage (> 3.20%) in the previous three bulk tank milk samples collected by the milk processor.

A sample of milk was collected from the bulk tank at the time of each individual farm visit. Milk in bulk tanks was initially cooled to between 0 °C and 4.4 °C and subsequently agitated for 10 min prior to sample collection. After opening the bulk tank valve, the first two litres of milk were discarded. Subsequently, one litre was collected from the bulk tank in a jug, from which a 120 ml subsample was taken. Milk samples were preserved immediately (Broad Spectrum Microtabs II, D&F Control Systems Inc., Norwood, MA). Within 24 h after collection, milk samples from selected herds were frozen at -20 °C and subsequently analysed in the university laboratory for milk fat percentage by wet chemistry according to the Gerber method [59]. Concentrations of milk protein, milk lactose, milk SCC, milk urea nitrogen (MUN), and milk casein were determined in a commercial milk laboratory (National Milk Laboratories Ltd., Wolverhampton, UK) using mid-infrared spectrometry (Milko-Scan FT6000, Foss Electric, Hillerød, Denmark; [60]. Cheddar cheese yield was calculated as [(0.93 × milk fat percentage) + (milk casein percentage − 0.1) × 1.09] / (1 − moisture content); [61], where cheese moisture content was assumed to be 35% [62].

Herds were then allocated to one of three categories based on the results using the Proc Univariate procedure of SAS 9.4. Herds selected for the study had milk fat percentages ranging from 2.60% to 4.50% (SD = 0.45). The top 25% (*n* = 24) of herds were categorized as high milk fat (HMF > 3.95% milk fat), the middle 50% (*n* = 42) of herds were categorized as mid milk fat (MMF = 3.31 – 3.94% milk fat), and the bottom 25% (*n* = 28) were categorized as low milk fat (LMF < 3.30% milk fat). The average milking herd size (number of cows milked on the day of sampling) of the herds enrolled in the study was 137 ± 109 cows (mean ± SD) (range 17 to 580 cows) with an average daily milk yield of 25.6 L ± 3.6 L/cow (mean ± SD) (range 17.0 to 36.0 L) at the time of sampling. The mean milk fat % of the HMF herds was 4.18% (3.95 to 4.50%; SD = 0.15), mean fat % for MMF herds was 3.61% (3.35 to 3.90%; SD = 0.17) and mean milk fat % for the LMF herds was 3.08% (2.60 to 3.30%; SD = 0.20). Assessment of herd diets was performed by recording the quantity of concentrate fed, and by sampling the pasture allocation that the herd would graze immediately post the farm visit. A farm management survey was completed by the farmer at the time of the farm visit. Details recorded in the survey were based on information provided by the farmer at the time of the visit. Total details collected in the survey included: number of milkings in bulk tank, estimated pre-grazing herbage mass of previous paddock (kg DM/ha), pasture allocation per cow per day (kg DM/cow/day), percentage of paddocks reseeded in previous 10 years, and concentrate feeding rate (kg/cow/day). Pre-grazing herbage mass of the next grazing allocation was determined by the cut and weigh method, using a quadrat (0.25 m^2^) and shears [63, 64]. The quadrat was tossed 3–4 times at random locations across the next grazing allocation, that were representative of the pre-grazing herbage mass. Subsequently, the area (0.25 m^2^) of the quadrat was cut using a handheld shears (Bosch Isio shears, Bosch GmbH, Gerlingen, Germany) to a height of 4 cm. This sample of pasture was weighed, and a sub-sample of pasture was taken for DM and chemical analysis. Average pre-grazing herbage mass for year 2 (not measured in year 1) was 1,862 ± 574 kg of DM/ha. Pasture and concentrate samples were collected from each farm and placed in separate resealable plastic bags at the time of each farm visit. Concentrate samples were frozen at -20 °C for analysis of chemical composition and FA composition, at a future date. Pasture samples were refrigerated initially at 4 °C, and oven dried within 24 h of collection. Likewise, pasture samples were stored in sealed plastic bags and subsequently analysed for chemical composition and FA composition. A forced-air oven was used to dry samples of pasture and concentrates at 55 °C for 72 h. The subsequent dried samples were ground using a Norris hammer mill fitted with a 1 mm screen (Christy and Norris Hammer Mill, Chelmsford, England). Concentrations of NDF and ADF were determined according to the method of [65] using the Ankom 220 Fiber Analyzer (Ankom™ Technology, Fairport, NY, US). Content of DM was determined by drying samples at 105 °C for 16 h [66]. Ash content was determined by incineration of a 5 g sample in a muffle furnace (Nabertherm GmbH, Lilienthal, Germany) at 550 °C for 5 h [67]. Starch content was determined using the Megazyme Total Starch Assay Procedure (product no: K-TSTA; Megazyme International Ireland Ltd, Wicklow, Ireland). Nitrogen content of the feed was determined by combustion using a Leco FP 528 instrument (Leco Instruments UK, Cheshire, UK) from this crude protein was estimated using N × 6.25 [68]. Ether extract (EE) was measured using a Soxtec instrument (Tecator, Höganäs, Sweden) and light petroleum ether according to the method of AOAC 107 [69]. The concentration of WSC was determined as described by [70].

Milk fat was extracted using the method of Folch [71] while methylation of FA was performed according to [72]. Fatty acid methyl esters (FAME) were quantified using a gas chromatograph (GCFID system Agilent 7890; Agilent, Santa Clara, CA, US) equipped with flame ionization detector (FID) and a CP-sil 88 fused silica capillary column for FAME (100 m × 0.25 mm [i.d.] with 0.2-μm film thickness), using helium as the carrier gas at a rate of 1 mL / minute. The initial temperature of 50 °C was held for 4 min after which it was increased to 110 °C at a rate of 8 °C per minute. Temperature was then increased at a rate of 5 °C per minute until it reached 170 °C, at which it was held for 10 min and then increased at a rate of 2 °C per minute until it reached 225 °C, which was then held for 30 min. This resulted in a total analysis time of 64 min. FAME Methyltricosanoate (C23:0-Methyl ester) was used as an internal standard. A reference standard (Supelco 37 Component FAME Mix) was used to determine recoveries and correction factors for individual FA as well as acting as a reference sample for routine quality control. Both reference and internal standards were purchased from Sigma-Aldrich (Arklow, Co. Wicklow, Ireland).

Extraction and methylation of the FA were carried out simultaneously according to the method of [73]. Esterification of FA was performed using a toluene and methanolic hydrochloric acid solution as follows: heating at 90 °C in a water bath for 2 h, cooling at room temperature and adding 1 mL of hexane and 10 mL of K_2_CO_3_ (6% *w/v*) and centrifuging for 5 min at 700 g. The organic solvent was thoroughly mixed with 1 g sodium sulfate and 1 g charcoal and centrifuged again. The solvent layer was immediately evaporated in a nitrogen stream to obtain an oily residue and dissolved in 0.8 mL of hexane. FAME from forage were separated, identified, and quantified using a gas chromatograph (GCFID system Agilent 7890; Agilent, Santa Clara, CA, US) using the same method as for milk.

The pH of milk samples was measured using a portable pH meter (Phoenix Instrument EC-25 pH/Conductivity Portable Meter). The RCT was determined by modification of the method by [74]. Approximately 5 mL of Hansen's Naturen 145 rennet (Chr. Hansen Holding A/S, Hørsholm, DK) was diluted with 100 mL of distilled water to give a 1/20 rennet dilution. A test tube containing 5 mL milk was placed in a water bath to allow a 5 min equilibrium time to reach 30 °C. Once the samples had reached 30 °C, 0.5 mL of the rennet dilution was added, and the timer started simultaneously. The sample was slowly inverted twice, attached to a rotating holder and immersed in the water bath at a 30° angle with rotation set to maximum speed (4 rpm). The length of time taken for milk to coagulate was recorded. The milk ethanol stability test has previously been applied as a surrogate for milk heat stability [75]. The ethanol stability was determined using the method reported by [76], previously described by [26]. Briefly, equal volumes of the milk were mixed with an ethanol solution (ranging in concentration from 62 to 84%, v/v) at room temperature. The ethanol stability of milk was determined at the maximum concentration of ethanol solution that did not cause milk coagulation.

### Statistical analysis

Data for all variables were screened for outliers before statistical analysis. Any data points more than three standard deviations from the mean were removed, with the exception of milk fat percentage. Data residuals were checked for normality using the UNIVARIATE procedure in SAS (version 9.1.3; SAS Institute, 2013). All data fitted the assumption of normality, and no transformations were required. Data were analysed as a completely randomized design using the MIXED procedure of SAS 9.4. The model included the fixed effects of classification (HMF, MMF or LMF), number of milkings in bulk tank, estimated pre-grazing herbage mass of previous paddock (kg DM/ha), grass allocation per cow per day (kg DM/cow/day), percentage of paddocks reseeded in previous 10 years, and concentrate feeding rate (kg/cow/day). Fixed effects were retained in the model if *P* < 0.10. All data presented in the manuscript are expressed as least squares differences ± standard error of the difference, unless otherwise stated. Statistical significance was declared at *P* < 0.05 and a tendency was assumed at *P* > 0.05 and < 0.10. Pearson correlation coefficients presented were determined using the CORR procedure of SAS for the Entire group and HMF, MMF and LMF groups.

## Data Availability

The datasets used and/or analysed during the current study are available from the corresponding author on reasonable request.

## Abbreviations

FA: Fatty Acid
MFD: Milk Fat Depression
HMF: High Milk Fat
LMF: Low Milk Fat
MMF: Mid Milk Fat
RCT: Rennet Coagulation Time
CLA: Conjugated Linolenic Acid
NDF: Neutral Detergent Fibre
WSC: Water Soluble Carbohydrate
MUFA: Monounsaturated Fatty Acid
PUFA: Polyunsaturated Fatty Acid
VFA: Volatile Fatty Acid
LNA: Linolenic Acid
UFA: Unsaturated Fatty Acid
TMR: Total Mixed Ration
MUN: Milk Urea Nitrogen
ADF: Acid Detergent Fibre
EE: Ether Extract
FAME: Fatty Acid Methyl Esther
